# CSE1L interaction with MSH6 promotes osteosarcoma progression and predicts poor patient survival

**DOI:** 10.1038/srep46238

**Published:** 2017-04-07

**Authors:** Dong-dong Cheng, He-chun Lin, Shi-jie Li, Ming Yao, Qing-cheng Yang, Cun-yi Fan

**Affiliations:** 1 Department of Orthopedics, Shanghai Jiao Tong University Affiliated Sixth People’s Hospital, Shanghai, 200233, China; 2 State Key Laboratory of Oncogenes and Related Genes, Shanghai Cancer Institute, Renji Hospital, Shanghai Jiao Tong University School of Medicine, Shanghai, 200032, China

## Abstract

To discover tumor-associated proteins in osteosarcoma, a quantitative proteomic analysis was performed to identify proteins that were differentially expressed between osteosarcoma and human osteoblastic cells. Through clinical screening and a functional evaluation, chromosome segregation 1-like (CSE1L) protein was found to be related to the growth of osteosarcoma cells. To date, little is known about the function and underlying mechanism of CSE1L in osteosarcoma. In the present study, we show that knockdown of CSE1L inhibits osteosarcoma growth *in vitro* and *in vivo*. By co-immunoprecipitation and RNA-seq analysis, CSE1L was found to interact with mutS homolog 6 (MSH6) and function as a positive regulator of MSH6 protein in osteosarcoma cells. A rescue study showed that decreased growth of osteosarcoma cells by CSE1L knockdown was reversed by MSH6 overexpression, indicating that the activity of CSE1L was an MSH6-dependent function. In addition, depletion of MSH6 hindered cellular proliferation *in vitro* and *in vivo*. Notably, CSE1L expression was correlated with MSH6 expression in tumor samples and was associated with poor prognosis in patients with osteosarcoma. Taken together, our results demonstrate that the CSE1L-MSH6 axis has an important role in osteosarcoma progression.

Osteosarcoma is the most common malignant bone tumor in adolescents^1^. With the implementation of comprehensive treatments, including chemotherapy and surgery, the outcome of patients with osteosarcoma has significantly improved in the past few decades. Currently, the 5-year survival rate is approximately 70%^2^. Although extensive research and investigation have significantly improved our understanding of osteosarcoma tumorigenesis, there are many patients who are insensitive to chemotherapy and have a poor prognosis. Therefore, there is an urgent need to identify new therapeutic targets.

Mass spectrometry-based quantitative proteomics is a powerful tool for the discovery of tumor-related genes and therapeutic targets^3^, in particular, isobaric tags for relative and absolute quantification (iTRAQ) labeling followed by nano liquid chromatography-mass spectrometry (NanoLC-MS/MS), to investigate tumors in various cancers such as liver cancer^4^, gastric cancer^5^, and oral cavity squamous cell carcinoma^6^. In addition, quantitative proteomic techniques are widely used in the study of osteosarcoma. To identify new biomarkers for a more accurate diagnosis of osteosarcoma, a proteomic approach was used to compare osteosarcoma cells and human primary cultured osteoblasts^7^. The authors found that 8 proteins, including HSP70, UQCRC1, and PRDX4, were increased in osteosarcoma and therefore may be potential molecular targets for osteosarcoma diagnosis and therapy. Similarly, another study performed a comparative proteomic analysis of human osteosarcoma and benign bone tumors and found that 12 proteins were upregulated and 6 proteins were downregulated^8^. The authors concluded that aberrant expression of cytoskeletal- and microtubule-associated proteins in osteosarcoma might promote tumor invasion and metastasis, which consequently may affect the prognosis of patients with osteosarcoma. Currently, in addition to identifying tumorigenesis-related genes, comparative proteomics can also be used for the identification of biomarkers that predict chemotherapy response in patients with osteosarcoma^9^. However, the function and underlying mechanism of the molecular targets discovered in these previous studies have not been well studied.

In the present study, a comparative proteomic analysis was performed on the osteosarcoma U2OS cell line and the human osteoblastic cell line hFOB 1.19. Through clinical analysis and functional evaluation, it was found that the human *CSE1L* gene on chromosome 20q13 was associated with osteosarcoma tumorigenesis. CSE1L is implicated in the regulation of multiple cellular mechanisms^10^ and is found in the cytoplasm and nuclei of cells. We found that the protein was located in the nuclei of osteosarcoma cells. CSE1L is highly expressed in several cancer types and positively correlates with tumor grade and poorer outcomes^11^. This present study demonstrated that knockdown of CSE1L inhibited osteosarcoma cell growth *in vitro* and *in vivo*. To investigate the underlying mechanism, we used co-immunoprecipitation and RNA-seq techniques and found that CSE1L interacted with and functioned through MSH6 in osteosarcoma cells. Analysis of clinical data revealed that CSE1L was overexpressed in osteosarcoma tissues compared to non-tumor tissues and that increased CSE1L expression in osteosarcoma was associated with a higher recurrence rate and poor survival. In addition, a positive correlation between CSE1L and MSH6 was found in osteosarcoma. Taken together, our findings demonstrate that *CSE1L* is an oncogene in osteosarcoma, and thus may be a potential target for osteosarcoma treatment.

## Results

### Comparative proteomics of osteoblastic cells versus osteosarcoma cells

**Figure 1:**
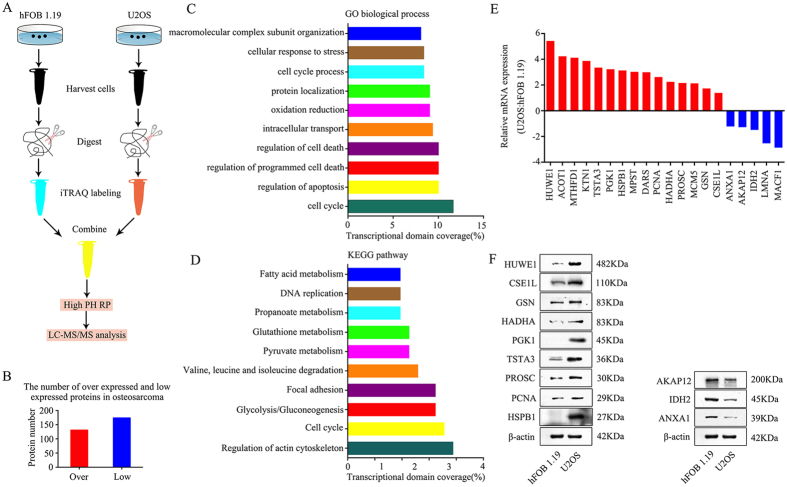
Experimental workflow for quantitative proteomic analysis and findings. (**A**) The flow chart for proteomic analysis. (**B**) The number of upregulated and downregulated proteins identified in osteosarcoma cells. (**C**) Gene ontology (GO) categories of overlapping genes. (**D**) Kyoto Encyclopedia of Genes and Genomes (KEGG) pathway analysis showing the top 10 enriched pathways. (**E**) mRNA expression levels of 15 upregulated and 5 downregulated proteins found in U2OS cells compared to those found in hFOB 1.19 cells as verified by qRT-PCR. (**F**) Representative blots displaying protein expression of 9 upregulated and 3 downregulated proteins. β-actin was used as an internal control. Full-length gels are presented in Supplementary Figure S7.

To determine differentially expressed proteins between osteoblasts and osteosarcoma cells, iTRAQ combined with NanoLC−MS/MS analysis was performed between hFOB 1.19 human osteoblasts and U2OS osteosarcoma cells. In total, 131 upregulated and 174 downregulated proteins were identified and quantified in U2OS cells compared to expression levels in hFOB 1.19 cells (Fig. 1B). We performed GO analysis and KEGG pathway analysis on these differentially expressed proteins. Based on the GO analysis, the main molecular functions identified were cell cycle, regulation of apoptosis, and regulation of programmed cell death (Fig. 1C), whereas the KEGG pathway analysis identified regulation of the actin cytoskeleton, cell cycle, and glycolysis/gluconeogenesis as the major pathways (Fig. 1D). According to PubMed data and our study, we divided the dysregulated proteins into three classes, including proteins that had been reported in osteosarcoma, proteins that had been reported in tumorigenesis but not in osteosarcoma and proteins that had not been reported in tumorigenesis. We could not validate every protein in this study because of the large number of dysregulated proteins. Finally, 20 potential osteosarcoma-associated proteins were selected to further validate the proteomic results. Among the 20 proteins, HUWE1, PCNA, GSN, ANXA1, AKAP12, and IDH2 have major roles in osteosarcoma tumorigenesis^12^,^13^,^14^,^15^,^16^,^17^. MTHFD1, TSTA3, PGK1, HSPB1, MPST, HADHA, MCM5, CSE1L, LMNA, and MACF1 have been reported to be involved in tumorigenesis but not in osteosarcoma^18^,^19^,^20^,^21^,^22^,^23^,^24^,^25^,^26^,^27^. ACOT1, KTN1, DARS, and PROSC have not been reported in tumorigenesis. As shown in Fig. 1E, the mRNA expression levels of these proteins, as measured using qRT-PCR, were consistent with those determined by the proteomic analysis. Because of limited antibodies and the large number of potential candidate proteins (20 proteins), we narrowed down our range of interested candidates to 12 proteins (including 9 upregulated proteins, HUWE1, CSE1L, GSN, HADHA, PGK1, TSTA3, PROSC, PCNA, and HSPB1, and 3 downregulated proteins, AKAP12, IDH2 and ANXA1) to detect the protein expression levels using western blotting. The protein expression levels of these proteins were consistent with those determined by the proteomic analysis (Fig. 1F). The expression of these 9 upregulated proteins was measured using qRT-PCR in 20 human tissue sample pairs in which each pair consisted of an osteosarcoma sample and a corresponding non-tumor tissue sample. The results revealed that mRNA expression of CSE1L was increased in osteosarcoma tissues than in the corresponding non-tumor tissues (Supplementary Figure S1). No differences in mRNA expression were found in the other 8 proteins. However, PCNA and GSN were reported to be overexpressed in osteosarcoma tissues compared with non-tumor tissues^13^,^14^. We believe that is because, in the present study, the number of samples for clinical screening was small (20 pairs) and because of the existence of tumor heterogeneity. Taken together, the results showed that CSE1L may have an important role in osteosarcoma tumorigenesis and thus warrants further investigation.

### Role of CSE1L in osteosarcoma cells *in vitro*

**Figure 2:**
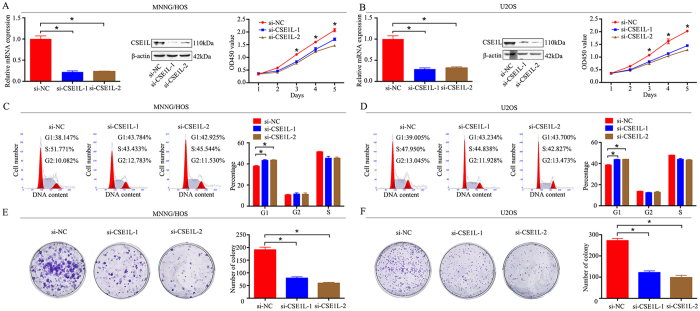
Knockdown of CSE1L inhibits osteosarcoma cell growth *in vitro*. (**A**,**B**) Levels of mRNA and protein expression were validated after CSE1L-specific siRNAs transfection in MNNG/HOS cells and U2OS cells by qRT-PCR and western blotting, respectively. Cell Counting Kit-8 (CCK-8) assays were performed after siRNA transfection. (**C**,**D**) Cell cycle assays were performed for CSE1L-silenced osteosarcoma cells and control cells. (**E**,**F**) Colony formation assays were performed for CSE1L-silenced osteosarcoma cells and control cells. Data are representative of results from three independent experiments. *P < 0.05. For western blotting, full-length gels are presented in Supplementary Figure S8.

To explore the functional significance of CSE1L in osteosarcoma, two individual CSE1L-specific siRNAs were used to knockdown CSE1L in two osteosarcoma cell lines, MNNG/HOS and U2OS. CSE1L knockdown was validated using qRT-PCR and western blotting. CSE1L mRNA and protein expression were significantly reduced after transfection with CSE1L-specific siRNAs in MNNG/HOS and U2OS cells (Fig. 2A and B). Next, a CCK-8 assay was used to detect cell proliferation, and we found that knockdown of CSE1L significantly inhibited the growth of osteosarcoma cells (Fig. 2A and B). Change to the cell cycle following CSE1L knockdown was analyzed by flow cytometry. The results from the cell cycle assay showed that knockdown of CSE1L delayed cell cycle progression by inhibiting the G1 phase transition (Fig. 2C and D). Similarly, findings from the colony formation assay showed that knockdown of CSE1L attenuated the formation of cell colonies (Fig. 2E and F). In addition, the cell apoptosis assay showed that knockdown of CSE1L promoted the cell apoptosis in osteosarcoma cells (Supplementary Figure S2). However, the results from migration and invasion assays showed that CSE1L knockdown did not affect migration or invasion of osteosarcoma (Supplementary Figure S3). These results demonstrate that CSE1L has an oncogenic role in osteosarcoma.

### Knockdown of CSE1L inhibits tumor growth *in vivo*

**Figure 3:**
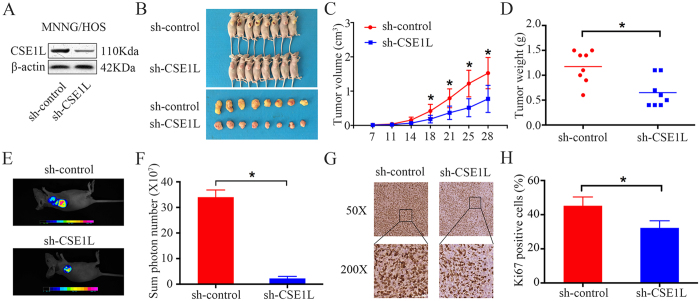
CSE1L knockdown inhibits osteosarcoma cell growth *in vivo*. (**A**) Representative blots displaying CSE1L protein expression in MNNG/HOS cells stably expressing sh-control or sh-CSE1L. β-actin was used as an internal control. (**B**) The upper diagram shows a photograph of tumor-bearing mice; the lower panel shows a photograph of the tumors when the mice were euthanized. (**C**) Growth curve drawn by measuring tumor volumes on the indicated days. Error bars represent the SEM. (**D**) This diagram shows the tumor weight in the sh-control group and the sh-CSE1L group. (**E**,**F**) These diagrams show representative green fluorescent protein (GFP) imaging and average photon number of the sh-control group and the sh-CSE1L group. (**G**,**H**) Representative images of Ki-67 staining in the sh-control group and the sh-CSE1L group. Magnification: 50×, 200×. For western blotting, full-length gels are presented in Supplementary Figure S9.

To further determine the effect of CSE1L on osteosarcoma growth *in vivo*, MNNG/HOS cells stably expressing sh-control or sh-CSE1L were constructed. Protein expression was validated by western blotting (Fig. 3A). Transfected cells were subcutaneously injected into the left scapulae of nude mice, and the animals were closely monitored for tumor growth for 4 weeks. The tumor growth curve demonstrated that CSE1L knockdown significantly inhibited tumor growth *in vivo*. Both tumor volume and weight were reduced in the CSE1L knockdown group compared to that in the scramble control group (Fig. 3B,C and D). Following euthanization of the mice, green fluorescent protein (GFP) imaging was performed using the small animal imaging system, and the sum photon number was calculated. Typical GFP imaging of the sh-control group and the sh-CSE1L group is shown in Fig. 3E. The average photon number in the sh-CSE1L group was smaller than that in the sh-control group (Fig. 3F). Our findings revealed that the fluorescence signal in sh-CSE1L mice was lower than that in sh-control mice. As shown in Fig. 3G and H, IHC analysis identified that staining of the tissue proliferation marker Ki-67 was decreased in sh-CSE1L tumors than in sh-control tumors. Taken together, these results indicate that CSE1L knockdown hinders tumorigenesis of osteosarcoma cells *in vivo*.

### CSE1L interacts with MSH6 in osteosarcoma cells

**Figure 4:**
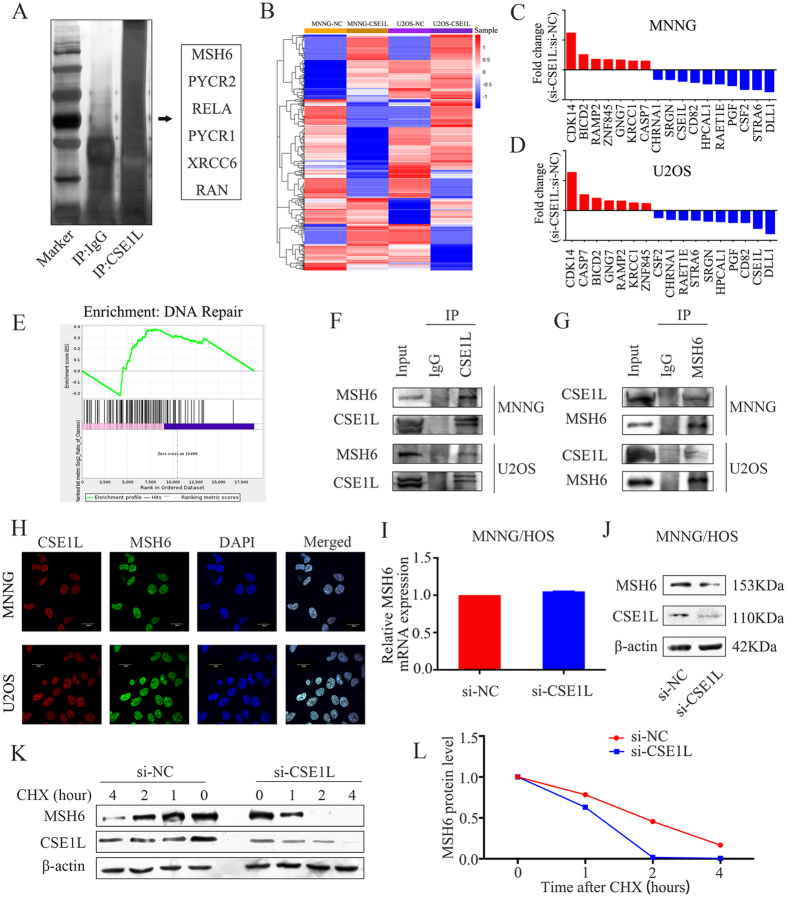
CSE1L interacts with MSH6 and affects its stability in osteosarcoma cells. (**A**) Silver staining of SDS-PAGE gel after IgG or CSE1L pulldown. (**B**) Heat map of differentially expressed genes between the si-NC-treated group and the si-CSE1L-treated group in osteosarcoma cells. (**C**,**D**) mRNA expression levels of 7 upregulated and 10 downregulated genes selected for validation using qRT-PCR. (**E**) Gene Set Enrichment Analysis (GSEA) demonstrated enrichment of a gene signature associated with DNA repair. (**F**) Whole-cell lysates from osteosarcoma cell lines were immunoprecipitated with an anti-CSE1L antibody followed by immunoblotting (IB) with anti-MSH6 and anti-CSE1L antibodies. IgG was used as a negative control. (**G**) Whole-cell lysates from osteosarcoma cell lines were immunoprecipitated with an anti-MSH6 antibody followed by IB with anti-CSE1L and anti-MSH6 antibodies. IgG was used as a negative control. (**H**) Immunofluorescence analysis was performed using anti-CSE1L and anti-MSH6 antibodies. DAPI was used as a control for nuclear staining. (**I**,**J**) MNNG/HOS cells were transfected with si-NC or si-CSE1L for 48 h. The effect of CSE1L knockdown on MSH6 mRNA and protein expression in MNNG/HOS cells was evaluated by qRT-PCR and western blotting. β-actin was used as an internal control. (**K**,**L**) MNNG/HOS cells were transfected with si-NC or si-CSE1L for 48 h, followed by 0, 1, 2, and 4 h treatment with 100 mg/mL cycloheximide (CHX). Lysates were IB with an anti-MSH6 or anti-CSE1L antibody. MSH6 band intensity was normalized to β-actin, then normalized to controls (time = 0). For western blotting, full-length gels are presented in Supplementary Figure S10.

To determine the underlying mechanism of CSE1L in osteosarcoma, co-immunoprecipitation was performed in MNNG/HOS cells. Following protein identification using mass spectrometry, 6 proteins (MSH6, PYCR2, RELA, PYCR1, XRCC6, and RAN) were chosen for verification (Fig. 4A). Then, RNA-seq was performed after transfection of CSE1L-specific siRNA into cells to identify changes in gene expression. The heat map of dysregulated genes is shown in Fig. 4B. In MNNG/HOS and U2OS cell lines, 668 genes were found to be altered after si-CSE1L transfection, in which 7 upregulated and 10 downregulated genes were selected for validation using qRT-PCR (Fig. 4C and D). The Gene Set Enrichment Analysis (GSEA) demonstrated that enrichment of the gene signature was associated with DNA repair (Fig. 4E). Because MSH6 is a DNA repair-related gene, we pulled down CSE1L and probed for MSH6. We found that CSE1L specifically interacted with MSH6 as shown in the immunoprecipitation assay (Fig. 4F). Conversely, we pulled down MSH6 and probed for CSE1L and found that MSH6 and CSE1L interacted with each other in MNNG/HOS and U2OS cells (Fig. 4G). In addition, we confirmed using confocal analysis that MSH6 and CSE1L are co-localized in MNNG/HOS cells and U2OS cells (Fig. 4H). Therefore, CSE1L associates with MSH6 in osteosarcoma cells. Next, we examined whether MSH6 mRNA or protein levels were affected by CSE1L. Knockdown of CSE1L in MNNG/HOS cells failed to alter MSH6 mRNA expression but led to a significant decrease of MSH6 protein expression (Fig. 4I and J). To further determine whether CSE1L affects MSH6 protein stability, we measured the half-life of MSH6 protein using a cycloheximide (CHX) chase assay and found that CSE1L knockdown markedly decreased the half-life of MSH6 protein (Fig. 4K and L). To identify the potential mechanism by which CSE1L affects MSH6 protein stability, MG132 (100 μmol/L) was added to MNNG/HOS cells for 6 hours after transfection with si-NC or si-CSE1L for 48 hours. The results showed that CSE1L knockdown-mediated reduction of MSH6 could be inhibited by the proteasome inhibitor MG132. Thus, we demonstrated that CSE1L affected MSH6 protein stability via the proteasome pathway (Supplementary Figure S4). Consequently, these results support the conclusion that CSE1L interacts with MSH6 and functions as a positive regulator of MSH6 protein in osteosarcoma cells.

### Knockdown of CSE1L inhibits osteosarcoma cell proliferation through MSH6

**Figure 5:**
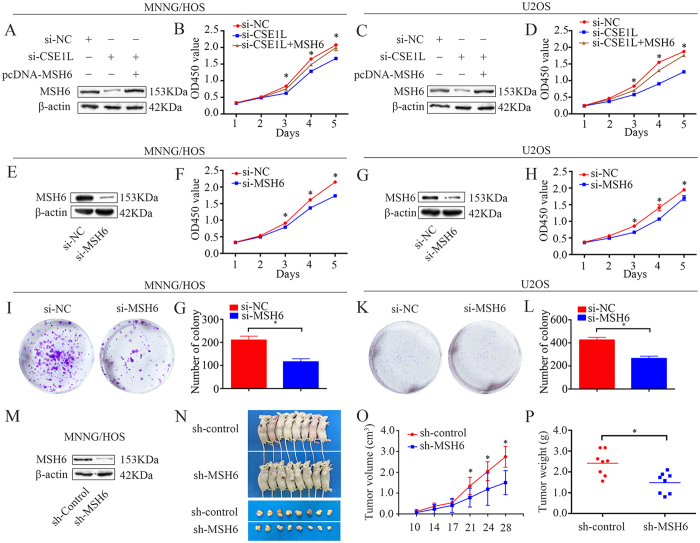
CSE1L knockdown inhibits osteosarcoma cell proliferation via MSH6. (**A**,**C**) Representative blots of MSH6 after transfection with pcDNA 3.1-MSH6, si-CSE1L or si-NC. β-actin was used as an internal control. (**B**,**D**) Cell Counting Kit-8 (CCK-8) assay was used to measure tumor cell proliferation after transfection with pcDNA 3.1-MSH6, si-CSE1L or si-NC. (**E**,**G**) Confirmation of protein expression levels following si-MSH6 transfection by western blotting in MNNG/HOS and U2OS cells. (**F**) (**H**) CCK-8 assay was performed to detect tumor cell proliferation after siRNA transfection. (**I**–**L**) Colony formation assays for MSH6-silenced osteosarcoma cells and control cells. (**M**) Representative blots displaying CSE1L protein expression in MNNG/HOS cells stably expressing sh-control or sh-MSH6. β-actin was used as an internal control. (**N**–**P**) Representative tumor-bearing mice, tumors isolated from nude mice, tumor volume, and tumor weights of an MNNG/HOS subcutaneous tumor model. Data are representative of results from three independent experiments. *P < 0.05. For western blotting, full-length gels are presented in Supplementary Figure S11.

Our findings demonstrated that knockdown of CSE1L inhibited osteosarcoma cell proliferation. However, the role of MSH6 in osteosarcoma is unknown, and whether CSE1L functions through MSH6 is largely unknown. Since CSE1L interacts with MSH6 and affects its protein expression and stability in osteosarcoma cells, it is plausible that CSE1L functions through MSH6 and that MSH6 has an important role in osteosarcoma cell proliferation. To test this hypothesis, we overexpressed MSH6 in CSE1L-knockdown MNNG/HOS and U2OS cells. Western blotting confirmed that MSH6 protein levels were rescued in CSE1L knockdown cells after pcDNA 3.1-MSH6 plasmid transfection (Fig. 5A and C); as hypothesized, MSH6 overexpression rescued the CSE1L knockdown-induced cell growth inhibition (Fig. 5B and D). Then, we detected the expression of MSH6 in osteosarcoma cells and osteoblastic cells. We found that MSH6 was highly expressed in osteosarcoma cells (Supplementary Figure S5A and B). Next, MSH6-specific siRNA was used to knockdown MSH6 in osteosarcoma cells. MSH6 knockdown was validated using qRT-PCR and western blotting (Supplementary Figure S5C and D, Fig. 5E and G). CCK-8 and colony formation assays were used to measure cell proliferation. We found that knockdown of MSH6 significantly inhibited the growth of osteosarcoma cells (Fig. 5F,H–L). To determine the role of MSH6 in osteosarcoma *in vivo*, we generated a stable MSH6-knockdown MNNG/HOS cell line. Western blotting confirmed that MSH6 protein expression was significantly reduced (Fig. 5M). Transfected cells were subcutaneously injected into the left scapulae of nude mice. The results revealed that both tumor volume and weight were reduced in the MSH6 knockdown group than in the scramble control group (Fig. 5N–P).

### CSE1L is a prognostic marker for osteosarcoma

**Figure 6:**
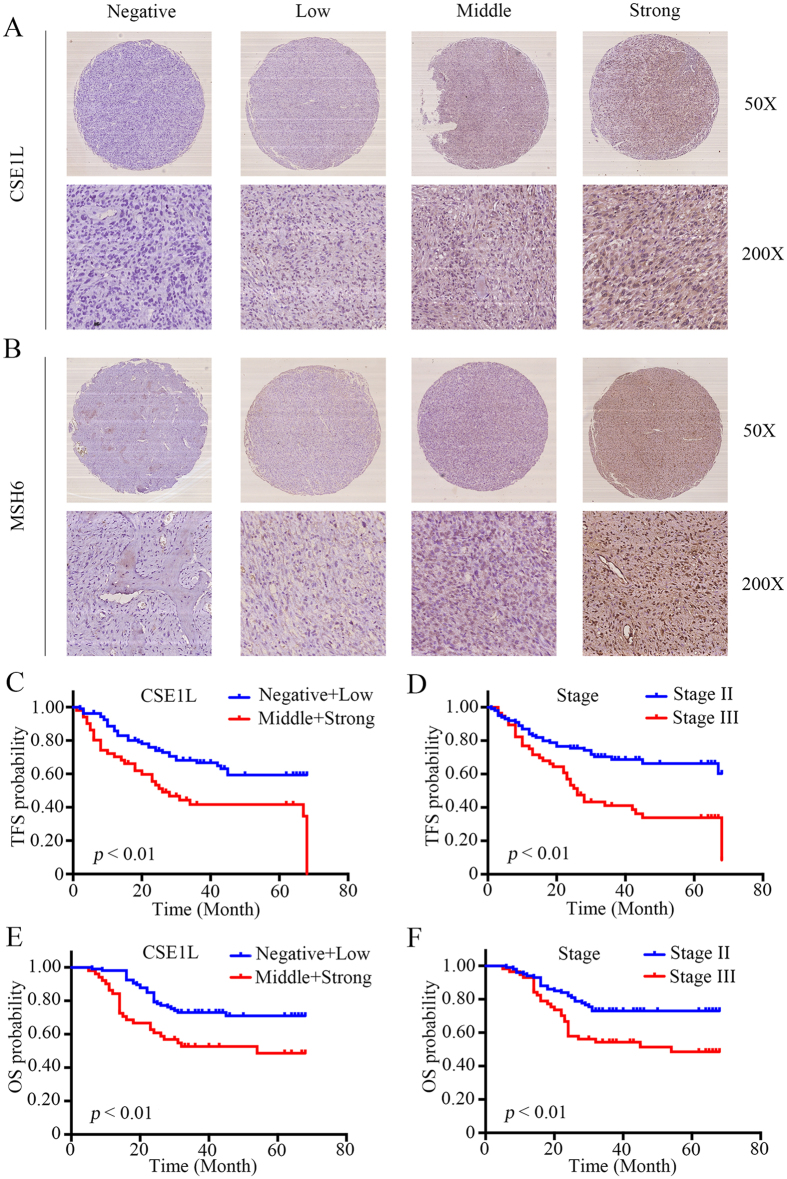
Clinical significance of CSE1L in patients with osteosarcoma. (**A**,**B**) Representative immunohistochemical (IHC) images of CSE1L and MSH6 expression in osteosarcoma tissues. IHC signal intensity scale: negative, low, middle, strong. Original magnification: 50×, 200×. (**C**–**F**) The effects of CSE1L expression level and Enneking stage on tumor-free survival and overall survival of patients with osteosarcoma.

To further determine the clinicopathological significance of CSE1L in osteosarcoma, we performed IHC analysis of CSE1L in a tissue microarray that includes an independent set of 157 cases of osteosarcoma. Representative IHC images of CSE1L expression are shown in Fig. 6A. Correlations between CSE1L expression level and clinicopathological characteristics of patients with osteosarcoma are summarized in Table 1. The expression level of CSE1L was higher in patients at a clinically advanced Enneking stage than at an early stage (*P* = 0.001). Further analysis found that CSE1L level was positively correlated with recurrence (*P* < 0.001), indicating that CSE1L has an important role in osteosarcoma recurrence. Univariate analysis showed that tumor-free survival (TFS) was related to CSE1L (*P* < 0.001) and Enneking stage (*P* < 0.001). Overall survival (OS) was also related to CSE1L (*P* = 0.005) and Enneking stage (*P* = 0.006) (Table 2). Variables that were identified as significantly different in univariate analysis were used for multivariate analysis. The Cox proportional hazards model showed that CSE1L (χ^2^ = 7.797, HR = 1.358, *P* = 0.005) and Enneking stage (χ^2^ = 6.031, HR = 1.898, *P* = 0.014) were independent prognostic variables for TFS (Table 3). In addition, the Cox proportional hazards model showed that CSE1L (χ^2^ = 4.236, HR = 1.283, *P* = 0.040) and Enneking stage (χ^2^ = 4.030, HR = 1.779, *P* = 0.045) were independent prognostic variables for OS (Table 3). The OS and TFS curves for this cohort are presented in Fig. 6C,D–F. There was no evidence that any of the other factors, including gender, age, tumor location, tumor necrosis rate, and cortical destruction, significantly influenced prognosis. We also performed Kaplan-Meier survival analyses using microarray data (http://www. kmplot.com) from breast, lung, gastric and ovarian cancer patients. We found that CSE1L expression also correlated negatively with patient survival in other cancers, including breast cancer, gastric cancer and ovarian cancer (Supplementary Figure S6). Finally, we examined MSH6 expression in osteosarcoma tissues; representative images of MSH6 expression are shown in Fig. 6B. We found that there was a significant correlation between expression of both CSE1L and MSH6 in osteosarcoma tissues (R = 0.697, *P* < 0.001). Based on these findings, CSE1L is correlated with MSH6 in tumor samples and is associated with poor prognosis in patients with osteosarcoma.

## Discussion

Currently, osteosarcoma has a 5-year survival rate of approximately 70%. However, this rate has remained largely unchanged for the past three decades. Therefore, there is the urgent need to identify new therapeutic targets. Comparative proteomics is a powerful tool to screen and identify tumor-related proteins. In this study, iTRAQ quantitative proteomics was used to compare the proteome profile of osteosarcoma cells to that of human osteoblasts. One hundred and thirty-one upregulated and 174 downregulated proteins were identified in U2OS cells compared to the levels in hFOB 1.19 cells, many of which have been reported to be closely related to tumorigenesis. Twenty proteins that may be associated with osteosarcoma proliferation underwent proteomic validation using qRT-PCR, and 12 were verified by western blotting. It was reported that HADHA was a promising prognostic marker in renal cell carcinoma^28^ and that PGK1 was increased in radioresistant astrocytomas and therefore may promote radioresistance in U251 human glioma cells^29^. In addition, it was reported that TSTA3 was highly expressed in breast cancer tissues and cells and was correlated with poor survival. Furthermore, knockdown of TSTA3 decreased cell invasion and proliferation in breast cancer^19^. In the present study, we detected 9 proteins with upregulated expression in 20 paired osteosarcoma tissues and their corresponding non-tumor tissues using qRT-PCR. The results showed that CSE1L was overexpressed in osteosarcoma tissues, which indicates CSE1L may have an important role in osteosarcoma tumorigenesis.

Previous studies have found that CSE1L knockdown resulted in decreased cell proliferation, a reduction in colony formation in soft agar, and induction of apoptosis in colorectal cancer^30^. Yuksel *et al*.^31^ recently reported that cytoplasmic CSE1L expression was significantly correlated with axillary lymph node metastasis, a finding that demonstrated the critical role of CSE1L in breast cancer metastasis. However, Liao *et al*.^32^ reported that increased CSE1L expression did not increase cancer cell proliferation, CSE1L reduction inhibited metastasis in melanoma cells. These findings indicate that CSE1L may act as an oncogene in tumorigenesis. To date, the role of CSE1L and the underlying mechanism in osteosarcoma are largely unclear. To assess the effects of CSE1L in osteosarcoma cells, CCK-8, cell cycle, and colony formation assays were performed. In agreement with previous studies, we found that knockdown of CSE1L resulted in significant inhibition of cell growth *in vitro*. To clarify further the role of CSE1L in osteosarcoma proliferation, MNNG/HOS cells with stable CSE1L knockdown were injected into nude mice. The results demonstrated that CSE1L knockdown also dramatically impedes tumor growth *in vivo*.

Although several studies have investigated the role of CSE1L in tumorigenesis, few elucidated the underlying mechanism in depth. Lorenzato *et al*.^33^ reported that AKT activation drove the nuclear accumulation of CSE1L in ovarian cancer cells, which may affect transmission of pro-oncogenic signals. Li *et al*.^34^ reported that CSE1L was a target gene of miR-137 and that it had an important role in miR-137-mediated tumor suppression. Lorenzato *et al*.^35^ found that CSE1L protected ovarian cancer cells from apoptosis by regulating the expression of RASSF1C, an isoform of the pro-apoptotic gene RASSF1. In addition, it was reported that CSE1L regulated homologous recombination (HR) repair by interacting with RAD51, which is a key protein in the HR repair process^36^. In the present study, co-immunoprecipitation was used to examine the underlying mechanism of CSE1L in osteosarcoma. Using mass spectrometry, we identified several proteins, including MSH6, PYCR2, RELA, PYCR1, XRCC6, and RAN, that underwent further investigation. RNA-seq was performed after transfection of CSE1L-specific siRNA into cells, and Gene Set Enrichment Analysis (GSEA) demonstrated that enrichment of the gene signature was associated with DNA repair. Because MSH6 has a role in DNA repair^37^,^38^,^39^, it was selected for further investigation. In addition to presenting evidence supporting an interaction between CSE1L and MSH6, we found that CSE1L and MSH6 were co-localized in cells from two different osteosarcoma cell lines. Furthermore, it was demonstrated that CSE1L depletion decreased MSH6 protein levels and that CSE1L stabilized MSH6. This is the first study to demonstrate an interaction between CSE1L and MSH6 in osteosarcoma cells.

Many studies have reported on the role of MSH6 in tumorigenesis. The MSH6 protein is a protein of the DNA mismatch repair (MMR) system and is also referred to as a G/T-binding protein^40^. A previous study showed that increased MSH6 expression was significantly associated with an increased risk of melanoma mortality^41^. A similar result was found in patients with recurrent glioblastoma. Stark *et al*.^42^ reported that positivity for MSH6 was an indicator of reduced survival in patients with glioblastoma. Jentzsch *et al*.^43^ investigated the relationship between MSH6 expression and metastasis, response to chemotherapy, and survival time in patients with osteosarcoma. They found that patients with increased MSH6 expression had a poor prognosis. In the present study, to determine whether CSE1L functioned through MSH6, a rescue experiment was performed. We found that restoration of MSH6 protein expression rescued tumor cell growth and therefore demonstrated that CSE1L effect on cell proliferation was largely dependent on MSH6. In addition, MSH6 knockdown inhibited osteosarcoma cell proliferation *in vitro* and *in vivo*. Further validation showed that increased CSE1L expression in osteosarcoma was associated with poor prognosis and that there was a positive correlation between CSE1L and MSH6 in osteosarcoma. Consequently, findings from these previous studies and the converging lines of evidence from our present study strongly indicate that CSE1L is an important regulator of osteosarcoma progression.

Collectively, knockdown of CSE1L dramatically inhibits osteosarcoma cell growth *in vitro* and *in vivo*. More importantly, this study is the first to provide experimental evidence for the significance of the interaction between CSE1L and MSH6 in osteosarcoma cells, and reveals the molecular mechanism that may underlie CSE1L-mediated tumorigenesis. Furthermore, CSE1L expression may be a sensitive biomarker for prognosis of patients with osteosarcoma. Therefore, intervention via the CSE1L/MSH6 axis may be a feasible and effective strategy in the treatment of osteosarcoma.

## Methods

### Cell lines and cell culture

Two osteosarcoma cell lines (MNNG/HOS and U2OS) and one human osteoblastic cell line (hFOB 1.19) were used in this study. The cells were maintained at 37 °C in a humidified atmosphere containing 5% CO_2_. They were cultured in either Dulbecco’s Modified Eagle’s Medium (MNNG/HOS) or RPMI-1640 (U2OS) and supplemented with 10% fetal bovine serum (Biowest), 100 U/ml penicillin (Sigma-Aldrich) and 100 mg/ml streptomycin (Sigma-Aldrich). The hFOB 1.19 cell line was cultured according to established ATCC protocols.

### Proteomic analysis

Cells were cultured to 80% confluence and collected. All protein extraction procedures were performed on ice. Cell pellets were dissolved in a cell lysis buffer (Pierce) containing protease and phosphatase inhibitors and incubated on ice for 45 min. The lysate was centrifuged at 12,000× *g* for 15 min at 4 °C, and the supernatant was collected. iTRAQ labeling was conducted using an iTRAQ Reagent 4-Plex kit (Applied Biosystems) based on the manufacturer’s protocol^44^. One hundred micrograms of hFOB 1.19 or U2OS cell lysates was labeled with iTRAQ labeling reagents 114 or 115 (Applied Biosystems). After strong cation exchange and NanoLC−MS/MS analysis, protein identification and iTRAQ quantitation were performed using ProteinPilot4.1 software (AB SCIEX). Two physical replicates were performed. Figure 1A shows the flow chart of the proteomic analysis. Differentially expressed proteins underwent gene ontology (GO) analysis and Kyoto Encyclopedia of Genes and Genomes (KEGG) pathway analysis.

### RNA isolation and quantitative real-time PCR assays

Total RNA of human tissue samples and cultured cells was extracted with Trizol reagent (Invitrogen) and quantified using a NanoDrop 2000 (Thermo Fisher Scientific) according to the manufacturer’s respective protocol. cDNA was synthesized using a PrimeScript RT Reagent Kit (Takara). RT-PCR was performed with SYBR Green Premix Ex Taq (Takara) as follows: an initial denaturation step for 30 seconds at 95 °C followed by amplification with 40 cycles of 95 °C for 5 seconds and 60 °C for 20 seconds, concluding with a melting curve analysis. All reactions were performed in 10 μl reaction volumes in triplicate. Gene expression levels were measured using the comparative Ct method. Primer sequences are provided in Supplementary Table S1.

### Western blotting analysis

Lysates were extracted from cultured cells using a mixture of T-PER Protein Extraction Reagent (Thermo Fisher Scientific), PhosSTOP (Roche), and Complete Mini (Roche). Protein samples were separated using 6% or 8% sodium dodecyl sulfate-polyacrylamide gel electrophoresis (SDS-PAGE) and transferred to nitrocellulose membranes (Millipore). After blocking in 5% non-fat milk, the membranes were incubated with different primary antibodies, which are described in Supplementary Table S2. The secondary antibody (1:5000) was either an anti-rabbit IgG (Sigma-Aldrich) or an anti-mouse IgG (Sigma-Aldrich). Visualization was performed with SuperSignal West Femto Maximum Sensitivity Substrate (Thermo Fisher Scientific).

### Oligonucleotide transfection and stable cell line generation

Pre-designed siRNAs (Ribobio) and plasmids (Public Protein/Plasmid Library) were transfected into cells using Lipofectamine 2000 Reagent (Invitrogen) following the manufacturer’s protocol. For proliferation, cell cycle, migration, and invasion assays as well as RNA extraction and western blotting, cells were used 48 h after transfection. To construct stably transfected cell lines, scramble control, CSE1L-specific shRNA, or MSH6-specific shRNA lentiviral particles (Biotend) were used to infect cells according to the manufacturer’s protocol.

### Cell proliferation assays and cell cycle analysis

Cell proliferation assay: 48 h after transfection, 3000 cells were seeded into each well of a 96-well plate and incubated. A 10-μL aliquot of Cell Counting Kit-8 (CCK-8) (Dojindo) was added to triplicate wells, incubated for 2 h, and absorbance was measured at 450 nm. Each measurement was performed in triplicate, and experiments were repeated twice. Cell cycle analysis: 48 h after transfection, cells were fixed in 70% ethanol at −20 °C overnight. Cells were stained with 50 μg/mL propidium iodide (Kaiji) and analyzed using a FACSCalibur flow cytometer (BD Biosciences). The results were analyzed using ModFit software (BD Biosciences). Assays were independently performed three times.

### Colony formation assay

Forty-eight hours after siRNA transfection, MNNG/HOS or U2OS cells (1 × 10^3^ cells/well) were seeded in 6-well plates. After 10 days of incubation, cells in each well were fixed with 100% methanol for 30 min, stained with 0.1% crystal violet for 30 min, and cell colonies were counted. All assays were independently performed in triplicate.

### Animal experiments

All animal experiments were approved by the Ethics Committee of the Shanghai Jiao Tong University Affiliated Sixth People’s Hospital (YS-2016–064, 24 February 2016). All methods were performed in accordance with the relevant guidelines and regulations. For tumor growth assays, MNNG/HOS cells stably expressing sh-control, sh-CSE1L, or sh-MSH6 were injected subcutaneously into the left scapulae of nude mice (6-week-old BALB/c-nu/nu, 8 mice per group, 2 × 10^6^ transfected cells per mouse). Tumor volume was monitored twice a week and calculated using the formula: V = 0.5 × length × width^2^. After 4 weeks, mice were euthanized. For histological analysis, primary tumors were harvested at necropsy and fixed in 10% formalin. Fixed samples were embedded in paraffin, and three non-sequential serial sections were obtained per animal. For GFP imaging, a Berthold LB983 100 NC320 NightOwl System (Berthold)^45^ was used in the xenografts model to monitor the growth of MNNG/HOS cells.

### Co-immunoprecipitation and mass spectrometry protein identification

Co-immunoprecipitation was performed in MNNG/HOS and U2OS cells. Equal amounts of protein (3000 μg) were incubated with antibodies at 4 °C overnight followed by incubation with protein A/G magnetic beads (Biotool) for 3 h at 4 °C. The beads were washed using phosphate-buffered saline (PBS) containing 1% Triton X-100 and eluted using 2X protein loading buffer at 100 °C for 10 min. IgG-bound or CSE1L-bound proteins were separated using SDS-PAGE and stained with a silver staining kit (Beyotime Biotechnology). For mass spectrometry analysis, CSE1L-bound proteins were separated using SDS-PAGE and stained with Coomassie blue R250 (Solarbio). After destaining, reduction, and trypsin digestion for 12 h, the peptides were extracted using acetonitrile and analyzed using a NanoLC-2D Ultra system (Eksigent) equipped with a Triple TOF 5600 mass spectrometer (AB SCIEX). Protein identification was performed with ProteinPilot4.1 software (AB SCIEX). For this study, a strict unused confidence cutoff >1.3 and peptides ≥2 were used for protein identification.

### Confocal immunofluorescence

Confocal immunofluorescence was performed on MNNG/HOS and U2OS cells. Briefly, cells were fixed and incubated with a rabbit polyclonal anti-CSE1L antibody and a mouse monoclonal anti-MSH6 antibody overnight at 4 °C followed by 1 h incubation with secondary antibodies (Invitrogen). The cells were incubated with a 1:1000 dilution of 4′,6-diamidino-2-phenylindole (DAPI) for 5 min and viewed with a Fluoview FV1000 microscope (Olympus).

### Clinical samples and IHC

Twenty human tissue sample pairs in which each pair consisted of an osteosarcoma sample and a corresponding non-tumor tissue sample were obtained at Shanghai Sixth People’s Hospital. Total RNA was extracted for clinical screening. The tissue microarray contains 157 patients diagnosed with osteosarcoma at Shanghai Sixth People’s Hospital. They received primary surgical treatment and preoperative and postoperative neoadjuvant therapy. The median age was 18 years (range: 5–73 years). The follow-up period ranged from 24 to 68 months, and the median time was 45 months. Ethics approval was obtained from the Ethics Committee of the Shanghai Jiao Tong University Affiliated Sixth People’s Hospital, and written informed consent was obtained from each patient prior to sample collection. A statement to confirm that all methods were carried out in accordance with relevant guidelines and regulations. A standard IHC staining procedure was followed. Briefly, paraffin-embedded sections were cut at 4 μm, dewaxed in xylene, followed by heating in a microwave at 60 °C for 20 min in EDTA buffer (pH 9.0) for antigen retrieval. For each slide, endogenous peroxidase activity was blocked by a 10 min incubation in 0.3% H_2_O_2_ followed by incubation at 37 °C with a 1:100 dilution of the primary antibody (pAb) CSE1L (Proteintech), a 1:200 dilution of MSH6 (Abcam), or a 1:200 dilution of Ki-67 (DAKO). Slides were rinsed three times in PBS, incubated for 30 min with EnVision staining kit (DAKO), followed by three additional washes in PBS, and color was developed over 3–10 min in a moist chamber at room temperature using 3,3′-diaminobenzidine. Slides were counterstained in hematoxylin and dehydrated in a graded ethyl alcohol series (70%, 90%, and 100%). For sections used as negative controls, PBS substituted the primary antibody. Assessment of IHC staining was independently performed by two expert pathologists. Any discordance was resolved through discussion and consensus. IHC signal intensities were scored as follows: negative, low, middle, or strong. For Ki-67, the percentage of Ki-67-positive cells was determined.

### Statistical evaluation

Data were compiled and analyzed using SPSS version 21.0 (SPSS Inc., Chicago, IL, USA). Comparisons between different groups were performed using Chi-square tests. The independent prognostic significance of parameters was estimated using the Cox proportional hazards model, and correlation analysis between CSE1L and MSH6 was performed using the Spearman correlation test. The tumor-free survival time was defined as the period from surgery to the detection of new local lesions. The overall survival time was defined as the length of time between surgery and death. A *P* < 0.05 was considered significant.

## Additional Information

**How to cite this article**: Cheng, D.- *et al*. CSE1L interaction with MSH6 promotes osteosarcoma progression and predicts poor patient survival. *Sci. Rep.* **7**, 46238; doi: 10.1038/srep46238 (2017).

**Publisher's note:** Springer Nature remains neutral with regard to jurisdictional claims in published maps and institutional affiliations.

## Supplementary Material

Supplementary Information

## Figures and Tables

**Table 1:** CSE1L expression status n (%).

Clinicopathologic parameters	CSE1L expression level				*p* value
	Negative	Low	Middle	Strong	
Gender					0.266
Male	49	21	15	13	
Female	30	6	12	11	
Age (years)					0.105
<18	42	10	15	7	
≥18	37	17	12	17	
Location					0.521
Femur	40	12	14	16	
Tibia	25	8	9	7	
Elsewhere	14	7	4	1	
Tumor necrosis rate (%)					0.062
<90	63	15	22	19	
≥90	16	12	5	5	
Cortical destruction					0.206
Yes	64	26	24	19	
No	15	1	3	5	
Recurrence					0.000*
Yes	25	12	11	21	
No	54	15	16	3	
Metastasis					0.175
Yes	45	21	17	12	
No	34	6	10	12	
Enneking stage					0.001*
II	61	17	14	8	
III	18	10	13	16	

*P < 0.05.

**Table 2:** Impact of prognostic factors on TFS and OS by univariate analysis in osteosarcoma.

Clinicopathologic parameters	Tumor-free survival				Overall survival			
	NO.	HR	95% CI	*p*	NO.	HR	95% CI	*p*
Gender								
Male	98	1.300	0.850–2.194	0.198	98	1.356	0.792–2.320	0.267
Female	59				59			
Age (years)								
<18	74	1.540	0.946–2.507	0.082	74	1.142	0.667–1.953	0.629
≥ 18	83				83			
Location								
Femur	82	0.777	0.552–1.093	0.147	82	0.922	0.641–1.328	0.664
Tibia	49				49			
Elsewhere	26				26			
Tumor necrosis rate (%)								
<90	119	0.728	0.405–1.310	0.289	119	0.713	0.359–1.418	0.335
≥90	38				38			
Cortical destruction								
Yes	133	0.704	0.377–1.318	0.273	133	0.714	0.359–1.419	0.337
No	24				24			
CSE1L								
Negative	79	1.502	1.228–1.836	0.000*	79	1.384	1.104–1.734	0.005*
Low	27				27			
Middle	27				27			
Strong	24				24			
Enneking stage								
II	100	2.435	1.514–3.918	0.000*	100	2.120	1.242–3.617	0.006*
III	57				57			

*P < 0.05.

**Table 3:** Variables predictive of survival by COX proportional hazards model in osteosarcoma.

	Parametes	Wald χ^2^	Risk Ratio	95% CI	*P*
TFS	CSE1L	7.797	1.358	1.096–1.684	0.005*
	Enneking stage	6.031	1.898	1.138–3.166	0.014*
OS	CSE1L	4.236	1.283	1.012–1.627	0.040*
	Enneking stage	4.030	1.779	1.014–3.123	0.045*

*P < 0.05.
