# Supplementary material for: CSE1L interaction with MSH6 promotes osteosarcoma progression and predicts poor patient survival
Source: Sci Rep. 2017 Apr 7;7:46238. doi: 10.1038/srep46238 (PMC5384328; doi:10.1038/srep46238)
Supplement: Supplementary Information [file srep46238-s1.pdf]

CSE1L interaction with MSH6 promotes osteosarcoma progression and predicts poor patient survival

Dong-dong Cheng[1#], He-chun Lin[2#], Shi-jie Li[1], Ming Yao[2], Qing-cheng Yang[1]*, Cun-yi Fan[1]*

1. Department of Orthopedics, Shanghai Jiao Tong University Affiliated Sixth People's Hospital, Shanghai, 200233, China.

2. State Key Laboratory of Oncogenes and Related Genes, Shanghai Cancer Institute, Renji Hospital, Shanghai Jiao Tong University School of Medicine, Shanghai, 200032, China.

Methods:

Cell apoptosis assay

Forty-eight hours after transfection, the fluorescein isothiocyanate Annexin V Apoptosis Detection kit I (BD Pharmingen, San Diego, CA, USA) was used. Briefly, the cells were collected and centrifuged at 2000 × g for 5 min. Then the cells resuspended in 500 μl binding buffer, supplemented with 5 μl Annexin V and 5 μl propidium iodide (PI), for 15 min of dark treatment at the room temperature. The flow cytometry (FC500 MPL, Beckman Coulter, Brea, CA, USA) was used to analyze the samples.

In Vitro Migration and Invasion Assays

Cell migration and invasion assays were performed in a 24-well plate with 8-mm pore size chamber inserts (Corning, New York, NY, USA). For migration assays, after transfection with either si-NC or si-CSE1L, cells (5×10^4 cells/well) were transferred into the upper chamber of an uncoated membrane. For invasion assays, after transfection, cells (1×10^5 cells/well) were transferred into the upper chamber of a Matrigel-coated membrane. Cells were diluted using serum-free culture medium. In both assays, cells seeded into the upper chamber were suspended in 200 μl Dulbecco's modified Eagle's Medium (DMEM) without fetal bovine serum (FBS). The lower chambers contained 800 μL of DMEM with 10% FBS. Cells were incubated at 37 °C in 5% CO_2 for 12 h and 16 h for the migration and invasion assays, respectively. The membrane inserts were removed, and non-invading cells were removed from the upper surface of the membrane. Cells that had migrated to the

bottom of the chamber were fixed with 100% methanol for 30 min and stained with 0.1% crystal violet for 30 min. Finally, cells from a minimum of 10 random fields were imaged and counted using a CKX41 inverted microscope (Olympus, Tokyo, Japan). All assays were independently performed three times.

Supplementary Figures and supplementary Figure legends:

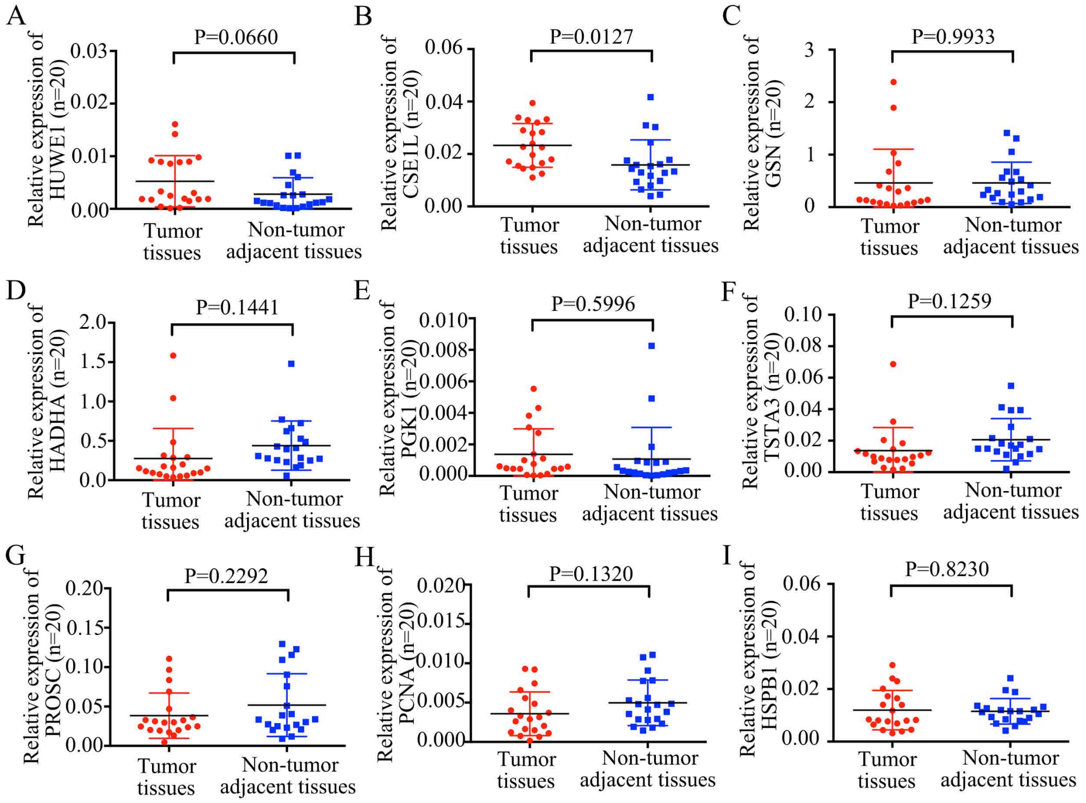

Supplementary Figure S1: The expression of these 9 upregulated proteins was measured using qRT-PCR in 20 human tissue sample pairs in which each pair consisted of an osteosarcoma sample and a corresponding non-tumor tissue sample.

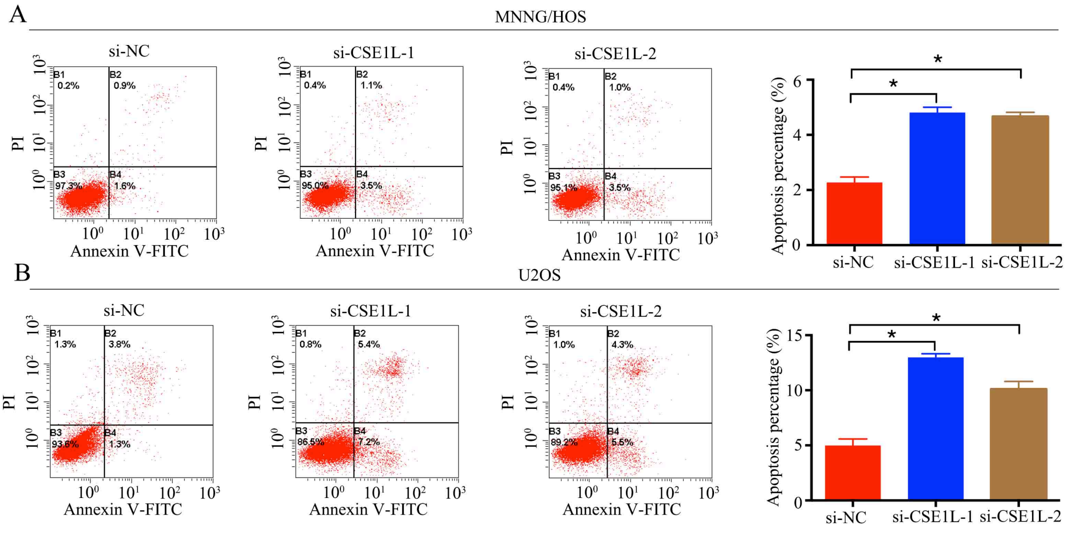

Supplementary Figure S2: Cell apoptosis assays were used to detect the cell

apoptosis after transfection with si-CSE1L in MNNG/HOS and U2OS cell lines. Statistical analysis was performed using Student's *t* test (n = 3). * *P*<0.05.

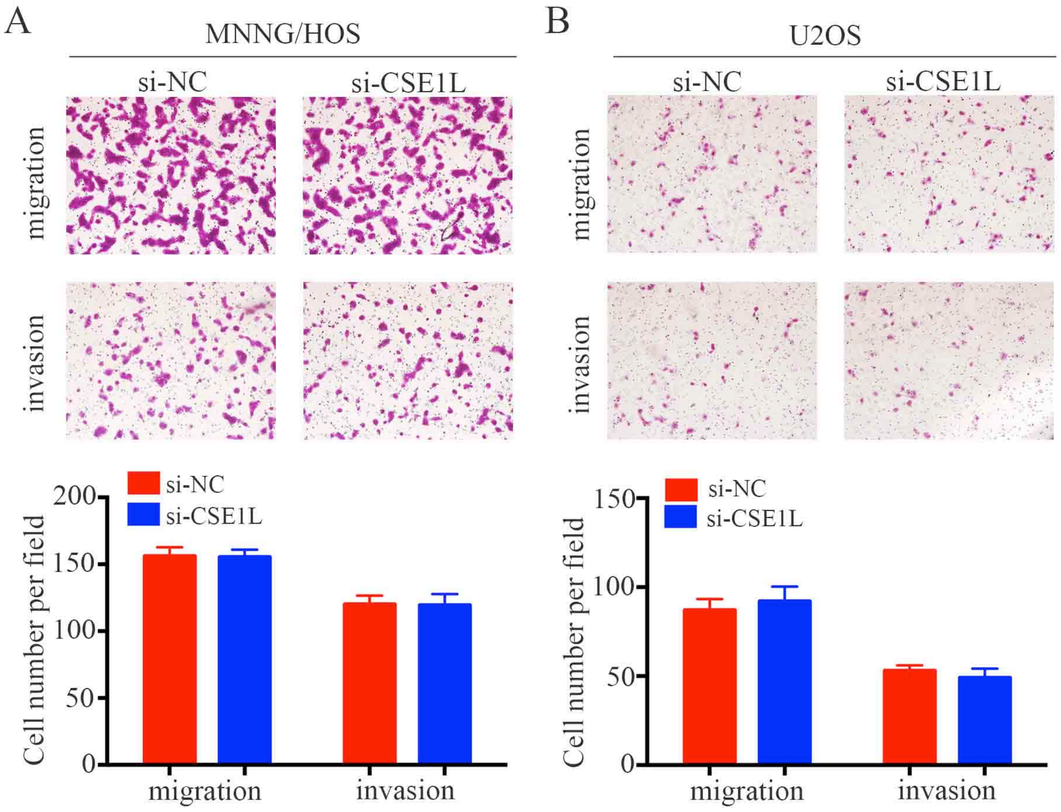

Supplementary Figure S3: Transwell assays were used to detect the migration and invasion capability of each cell line after transfection with si-CSE1L. The upper panels show representative photos (magnification: 100×) of invasive cells, and the lower panel shows histograms of the results. Statistical analysis was performed using Student's *t* test (n = 3).

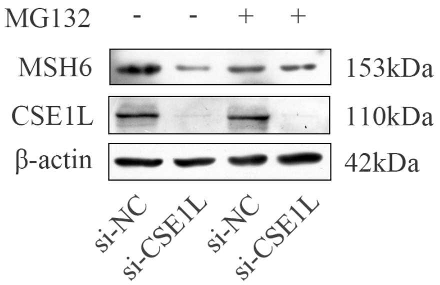

Supplementary Figure S4: CSE1L affected MSH6 protein stability via the proteasome pathway. Representative blots displaying protein expression of MSH6 treated with MG132 (100 μmol/L) or DMSO as a control for 12 h after transfection with si-NC or si-CSE1L. β-actin was used as an internal control.

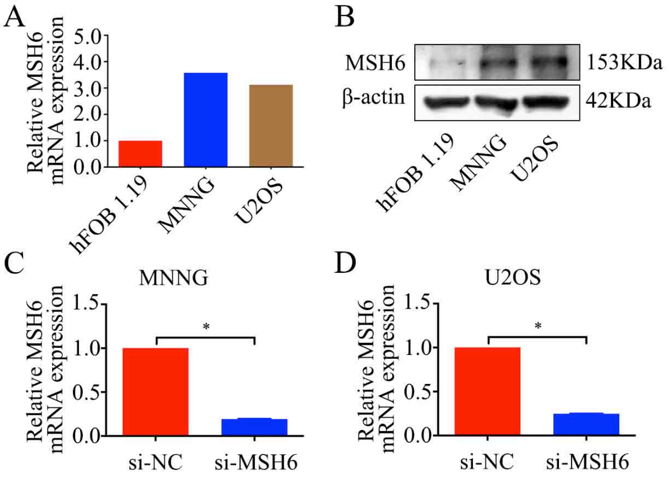

Supplementary Figure S5: (A) (B) The mRNA and protein expressions of MSH6 in osteosarcoma cells and osteoblastic cells were detected by qRT-PCR and western blotting. (B) The mRNA expression of MSH6 was detected after transfection with si-MSH6 in MNNG/HOS and U2OS cells. * *P*<0.05 by Student's *t* test.

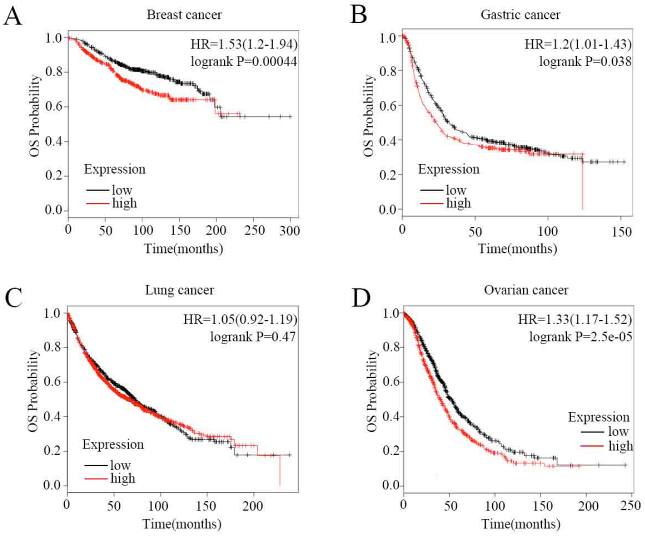

Supplementary Figure S6: Kaplan-Meier survival analyses were performed using

microarray data (http://www. kmplot.com) in breast cancer, lung cancer, gastric cancer and ovarian cancer patients.

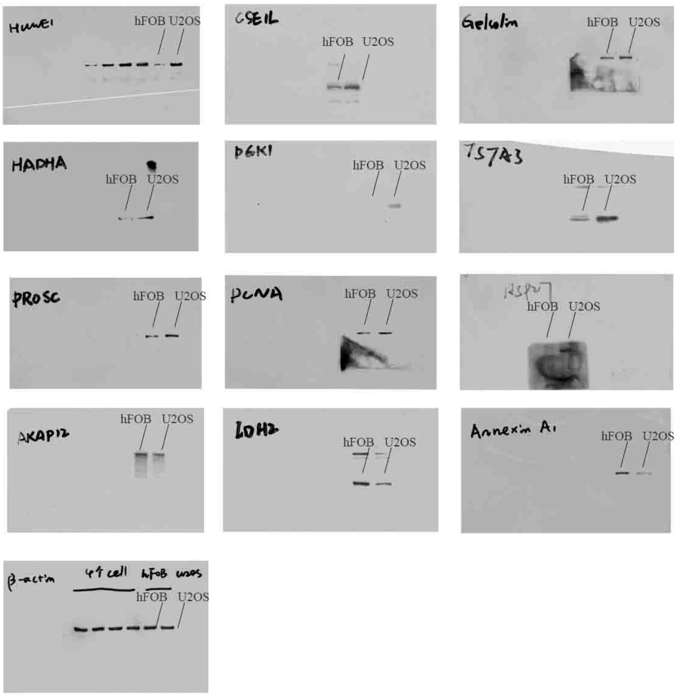

Supplementary Figure S7: Full-length gels for Figure 1

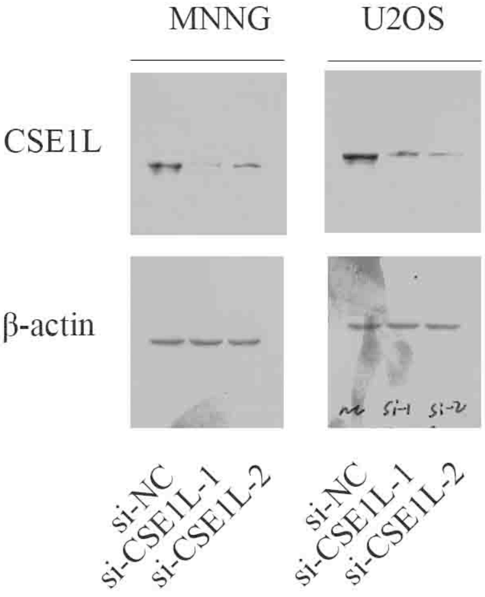

Supplementary Figure S8: Full-length gels for Figure 2

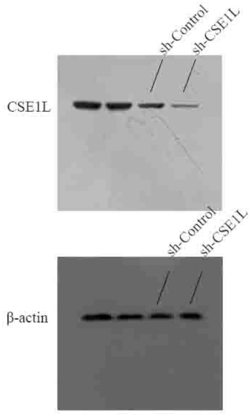

Supplementary Figure S9: Full-length gels for Figure 3

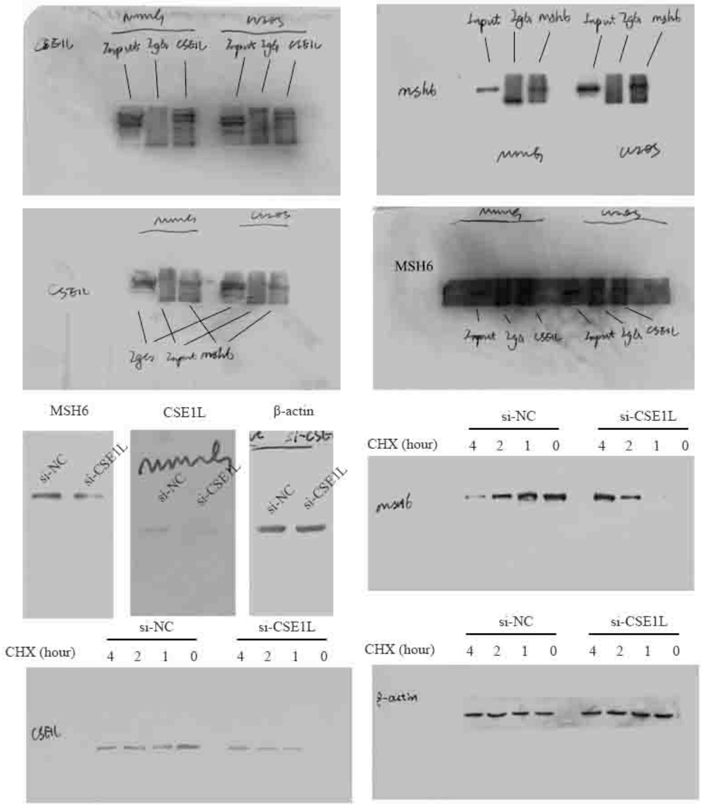

Supplementary Figure S10: Full-length gels for Figure 4

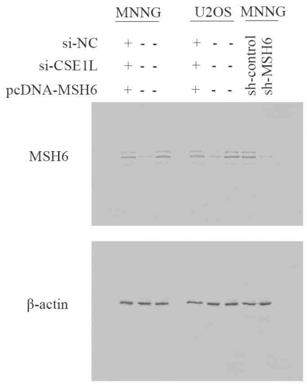

Supplementary Figure S11: Full-length gels for Figure 5

Supplementary Table S1 The primer sequences for qRT-PCR

Gene name		Sequence (5'->3')
GSN	Forward	ATGGAGACTTCTTCACGGGC
	Reverse	CACTCATTGCCCAGCCAGTA
ANXA1	Forward	AATCAGAAGCCCAAGTCTCCA
	Reverse	GGATGACTTCACAGTTTGAACAT
PROSC	Forward	CACCAGCGGAGAAGAGAGTAA
	Reverse	TCCTATGGTCATCAGCCCCA
IDH2	Forward	ACAACACCGACGAGTCCATC
	Reverse	GCCCATCGTAGGCTTTCAGT
CSE1L	Forward	GCAGCTCATGCTCTTGAACG
	Reverse	GCCAGGAAGTGTGAGAGCTT
AKAP12	Forward	GCTGGACAGGAAACGGAGAA
	Reverse	CACTGCGGTTGACTCTGACT
HUWE1	Forward	ACTGGTGCAACTTCCTCCTT
	Reverse	TTGTCCTGGGCTGCAATCTC
PCNA	Forward	TGAAGCACCAAACCAGGAGA
	Reverse	GTGCAAATTCACCAGAAGGCA
HADHA	Forward	AGACCTGAGAAGGTGATTGGC
	Reverse	GCGCAAGACACCTGGTAGTA
ACOT1	Forward	GCTGCTGTGCCGGGTG
	Reverse	CCCAGGTTCTGGCGGC
MTHFD1	Forward	ATGACCTCAAGCTCCCAGTTG
	Reverse	GCTTTGTGTTGAGCTTCGGG
KTN1	Forward	GTGAGCAGATGGAGGCAGAG
	Reverse	AGTTCTGCAGACTGCTTGCT
TSTA3	Forward	GGATGCTCCGTGCAACTG
	Reverse	GTGTGTGGGTTGGACCTTCT
PGK1	Forward	TGTAGGCCCAGAAGTGGAGA
	Reverse	CTGGCTCGGCTTTAACCTTG

HSPB1	Forward	CGCGGAAATACACGCTGC
	Reverse	CGGATTTTGCAGCTTCTGGG
MPST	Forward	CCGAGACGGCATTGAACCT
	Reverse	CTGAGATGACATCCTCGGGC
DARS	Forward	TCTCGCGATCTTTCTGGAGC
	Reverse	CCAAAACTCGATTTCCGCCG
MCM5	Forward	TCACCAAGCAGAAATACCCG
	Reverse	CGACTCACTTGAGGCGGTAG
LMNA	Forward	GCAGCATCATACAAGAGATGGG
	Reverse	CCTCCAGTGACTGCACAGAG
MACF1	Forward	AGACAAGTGGCTCAGTGCAA
	Reverse	GTTCTACCTCGTGCTCGGC
CDK14	Forward	GCTCGCCTCCCTAGACCT
	Reverse	TCTTCAAAGCAATGCGACTGA
BICD2	Forward	GCCACCAGGTGTGACGAGTA
	Reverse	TGTGACTTACGCTCGGTGTG
RAMP2	Forward	CTGTCCTGAATCCCCACGAG
	Reverse	CAGGGTGCTATAAGGCCTGC
ZNF845	Forward	GGATGGCTCTTTCTCAGGGTC
	Reverse	CCAGGGAGACCAGGTTCCTA
GNG7	Forward	GAGTGTCGGCCCCGC
	Reverse	AGCTTGCTGTACACCCTGTG
KRCC1	Forward	GCGGGGGACAGTAGTTGTAG
	Reverse	AACTGGTGAGCTCATAATGCAA
CASP7	Forward	GTGGGAACGATGGCAGATGA
	Reverse	TTCCGTTTCGAACGCCCATA
CHRNA1	Forward	TCTCCTCCTGCTCTTTAGCCT
	Reverse	CCCATTGCTGTTTCAGACGC
SRGN	Forward	ACTTCACGAGCTTGGCTCAG
	Reverse	TCTCCGCGTAGGATAACCTTG
CD82	Forward	GGCTGCTGAAGCAGGAGAT
	Reverse	CAGCACTTCACCTGAGCCTG
HPCAL1	Forward	CTGTGTTCGGGGCTGTCTGT

	Reverse	GCGGCGACTACACCAGAG
RAET1E	Forward	GACCTCAGTTGTTCCAGGGT
	Reverse	CTGTTGTACATGATCTCCAAGGC
PGF	Forward	TGTTCAGCCCATCCTGTGTC
	Reverse	AGCATCGCCGCACCTTTC
CSF2	Forward	CCCTCCAACCCCGGAAACTT
	Reverse	GCCATGCCTGTATCAGGGTC
STRA6	Forward	ACACTTCTCAGGGCCAACTG
	Reverse	CTGGCCCTTCTCCTCCAATC
DLL1	Forward	CCTGTTCTAATGGTGCCAAGTG
	Reverse	CACTCGCACACATAGCGGTG
MSH6	Forward	GGGATACAGCCTTTGACC
	Reverse	GTTTACAGCCCTTCTTGG
β-actin	Forward	TTGTTACAGGAAGTCCCTTGCC
	Reverse	ATGCTATCACCTCCCCTGTGTG

Supplementary Table S2 The primary antibody for Western blotting

Protein name	Dilution ratio	Reagent brand
HUWE1	1:500	Bioworld Technology
CSE1L	1:500	Proteintech
GSN	1:1000	Cell Signaling Technology
HADHA	1:200	Santa Cruz
PGK1	1:500	Proteintech
TSTA3	1:500	Proteintech
PROSC	1:1000	GeneTex
PCNA	1:1000	Proteintech
HSPB1	1:500	Proteintech
AKAP12	1:500	Proteintech
IDH2	1:1000	Epitomics
ANXA1	1:500	Proteintech
MSH6	1:200	Abcam
β-actin	1:20000	Sigma-Aldrich
